# A case of paraneoplastic myositis induced rhabdomyolysis due to pancreatic adenocarcinoma

**DOI:** 10.1093/omcr/omaf141

**Published:** 2025-08-20

**Authors:** Ariel Ahl, Rafi Orphali, Kumar Desai

**Affiliations:** Internal Medicine Residency Program, HCA Los Robles Health System, 227 West Janns Road Thousand Oaks, CA 91360, United States; Internal Medicine Residency Program, HCA Los Robles Health System, 227 West Janns Road Thousand Oaks, CA 91360, United States; Department of Gastroenterology/Hepatology, HCA Los Robles Health System, Thousand Oaks 91360, United States

**Keywords:** rhabdomyolysis, pancreatic cancer, paraneoplastic myositis

## Abstract

Rhabdomyolysis is a clinical syndrome characterized by the breakdown of skeletal muscle tissue, leading to the release of myoglobin and creatine kinase (CK) into the bloodstream. The condition clinically presents with myalgia, weakness, and dark urine. It can lead to kidney failure and can be life threatening. Various factors and conditions, including trauma, prolonged immobilization, strenuous exercise, infections, alcohol consumption, medications, and malignancies are potential causes. A rare cause of pancreatic cancer-induced paraneoplastic myositis has been observed. Herein, we present the case of an 87-year-old man who presented with fatigue, weight loss, and invasive pancreatic adenocarcinoma resulting in rhabdomyolysis due to paraneoplastic myositis.

## Introduction

Rhabdomyolysis is a potentially life-threatening condition that is caused by skeletal muscle breakdown. Its hallmark features include myalgia, weakness, and the release of intracellular content into the bloodstream. Complications include acute kidney injury (AKI), elevated CK levels, volume depletion, cardiac arrhythmias, disseminated intravascular coagulation, and acute renal failure [5]. The common causes include trauma, infection, exertion, prolonged immobilization, electrolyte abnormalities, drug use, and alcohol abuse [1, 5]. Paraneoplastic inflammatory myopathies, particularly dermatomyositis (DM) and polymyositis (PM), rarely lead to rhabdomyolysis [1]. PM is a paraneoplastic manifestation of non-Hodgkin’s lymphoma, as well as cancers of the bladder, breast and lung [3, 4]. However, in a few isolated cases, it has been seen to be associated with pancreatic cancer. This case report presents a rare occurrence of rhabdomyolysis in a patient with suspected paraneoplastic myositis due to pancreatic adenocarcinoma.

## Case presentation

**Figure 1:**
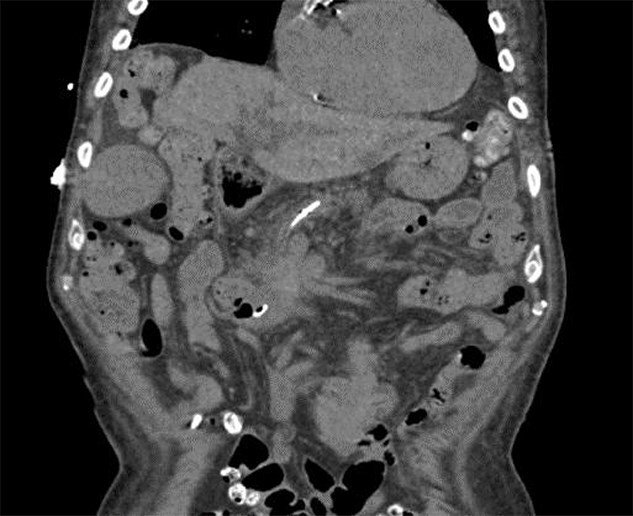
Computed tomography of the abdomen and pelvis demonstrating intact pancreatic duct stent.

An 87-year-old man with a history of hypothyroidism, hypertension, hyperlipidemia, and benign prostatic hyperplasia who presented to an outside hospital after an outpatient workup for fatigue, generalized weakness, jaundice, and 30-pound weight loss over three months revealed a pancreatic mass. The patient underwent endoscopic retrograde cholangiopancreatography (ERCP) in which a pancreatic duct stent was placed (Fig. 1). Unfortunately, free cannulation of the common bile duct (CBD) cannot be performed because of the presence of a stricture. The patient was transferred to our hospital.

On presentation, a physical examination revealed jaundice and scleral icterus. Laboratory values demonstrated BUN 52 mg/dl, Creatinine 3.45 mg/dl, total bilirubin 7.7 mg/dl, direct bilirubin 5.73 mg/dl, Aspartate Aminotransferase, 380 IU/l, Alanine Aminotransferase, 86 IU/l, and Alkaline Phosphatase, 1093 IU/l. Computed tomography of the abdomen and pelvis without intravenous contrast showed extensive intra and extrahepatic biliary ductal dilatation, a CBD 15 mm in caliber, and an appropriately positioned pancreatic duct stent. Gastroenterology was consulted for further management of the condition. Additional laboratory evaluation revealed a CK of 4400 IU/l and Myoglobin level of 226 ng/dl. Urinalysis demonstrated 3+ blood, RBC 1, WBC 0. Given these findings, there are concerns regarding rhabdomyolysis.

Subsequently, nephrological examination was performed. Treatment was initiated with intravenous fluid (IVF) and albumin administration.

Patient underwent endoscopic ultrasound (EUS) with ERCP, which revealed a dilated bile duct measuring 20 mm with a 3.4 cm abnormal-appearing pancreatic head mass and evidence of portal vein involvement. Biopsy of the mass was performed using fine-needle aspiration (FNA). ERCP was successful with the placement of a 10 × 80 Viabil stent and the removal of a previously placed pancreatic stent. An occlusion cholangiogram demonstrated a dilated bile duct with a distal 2 cm stricture. Biopsy results obtained via FNA revealed invasive pancreatic adenocarcinoma. Oncology was consulted and the possibility of palliative chemotherapy was discussed. After extensive discussion with the patient, he declined to pursue treatment and wished for hospice care.

Over the course of the hospital stay, his CK and myoglobin levels continued to decrease while receiving IVF and Albumin. His creatinine level continued to improve.

However, on day 8, the patient showed a rapid decline in his condition, which was noticeable along with worsening fatigue. CK and Myoglobin levels were significantly elevated (10 698 IU/L and > 40 000 IU/l, respectively). The patient’s renal function had declined. Aggressive management with IVF was continued until the patient was discharged with hospice therapy on Day 11.

## Discussion

Overall, inflammatory myopathies such as DM and PM are immune-mediated conditions that cause proximal and symmetric weakness. It primarily affects women (In contrast to our patient) [3]. These conditions, particularly DM, are associated with malignancies [4]. Paraneoplastic myopathies are most commonly observed in lung, breast, ovarian, colon, gastric, and Non-Hodgkin’s Lymphoma [3, 4]. However, there have been cases of pancreatic cancer, with several studies demonstrating this association with DM and only a few showing it with PM. Furthermore, based on the literature, only one case of rhabdomyolysis has been reported in pancreatic adenocarcinoma.

To highlight this, in a study by Ponyi et al., of the participants with PM, there was no cancer-related myositis, whereas it was observed in DM. Among those with cancer-related myositis, there were no associations with pancreatic adenocarcinoma [4]. A meta-analysis by Yang et al. demonstrated that patients with inflammatory myopathies have an increased risk of malignancy. Those with PM demonstrated a relative risk of 1.62 for overall malignancy, despite PM being observed with such an association, the exception for this did include pancreatic neoplasm [6]. However, several cases described by Kida et al., Syrios et al., Siddiqui et al., and Amroun et al. have demonstrated an association between PM and pancreatic adenocarcinoma in isolated cases [3].

Constantini et al. demonstrated that this association is complicated by rhabdomyolysis [1]. In their report, the patient, similar to ours, presented with progressively worsening lower extremity pain, fatigue, weakness, weight loss, and significant CK elevation.

The diagnosis of inflammatory myopathies typically involves muscle biopsy [1, 3]. Additional diagnostic, but not required, methods include electromyography (EMG) and autoantibodies. Other supportive measures include CK levels [3]. In the present case, although a complete work-up could not be obtained to confirm the diagnosis of paraneoplastic myositis given the patient’s declination for further treatment and wish for transition to hospice care, clinical suspicion remained high given the presentation and rapid deterioration. This could be supported by the elevated CK level, which suddenly worsened despite fluid treatment, along with worsening weakness and fatigue. This could also be supported by the lack of evidence for other causes of rhabdomyolysis.

Although no clear additional diagnostic methods have been elucidated, one case discussed the potential use of myositis-specific antibodies in the diagnosis of paraneoplastic inflammatory myositis [2].

Treatment of these inflammatory myopathies, whether paraneoplastic or idiopathic, involves high-dose corticosteroids, followed by transition to Azathioprine or Methotrexate. Responses to therapy can last from weeks to months; however, reports have noted that they can be less than expected [1, 3].

Among those complicated by rhabdomyolysis, treatment includes fluid resuscitation and management of the underlying cause. CK levels should be trended to ensure improvement in CK levels. More aggressive management involving hemodialysis should be considered in patients with oliguria or anuria with AKI despite fluid resuscitation [5]. In our case, despite the initial improvement in AKI and CK levels, the patient experienced an eventual rapid decline. This could potentially highlight that treatment or removal of the underlying cause may be essential in cases complicated by rhabdomyolysis, which we could not pursue.

For example, in the case of Padniewski et al., despite treatment with prednisone and chemotherapy, the patient died shortly thereafter. However, this was in contrast to those without rhabdomyolysis. For instance, in a report by Kida et al. reported that treatment with Prednisone, Fluorouracil, and radiotherapy resulted in a year of remission. In studies by Siddiqui et al. and Amroun et al., treatment involved tumor resection, which resulted in complete remission [3].

Based on the available literature and our case presentation, several key points have emerged regarding the intersection of inflammatory myopathies, rhabdomyolysis, and pancreatic adenocarcinoma. Increasing evidence indicates that inflammatory myopathies, including PM, are associated with pancreatic cancer.

## References

[1] Costantini A, Moletta L, Pierobon ES. et al. Paraneoplastic myopathy-related rhabdomyolysis and pancreatic cancer: a case report and review of the literature. World J Clin Cases 2023;11:6823–30. 10.12998/wjcc.v11.i28.6823 37901020 PMC10600837

[2] Lu X, Peng Q, Wang G. The role of cancer-associated autoantibodies as biomarkers in paraneoplastic myositis syndrome. Curr Opin Rheumatol 2019;31:643–9. 10.1097/bor.0000000000000641 31369431

[3] Padniewski JJ, Nelson E, Mian I. et al. Paraneoplastic myopathy in pancreatic cancer: a case report and literature review. J Community Hosp Intern Med Perspect 2021;11:847–51. 10.1080/20009666.2021.1982487 34804404 PMC8604545

[4] Ponyi A, Constantin T, Garami M. et al. Cancer-associated myositis: clinical features and prognostic signs. Ann N Y Acad Sci 2005;1051:64–71. 10.1196/annals.1361.047 16126945

[5] Stanley M, Adigun R. Rhabdomyolysis. PubMed. Published 2023. https://www.ncbi.nlm.nih.gov/books/NBK448168/

[6] Yang Z, Lin F, Qin B. et al. Polymyositis/dermatomyositis and malignancy risk: a Metaanalysis study. J Rheumatol 2014;42:282–91. 10.3899/jrheum.140566 25448790

